# A Wearable Electrocardiogram Telemonitoring System for Atrial Fibrillation Detection

**DOI:** 10.3390/s20030606

**Published:** 2020-01-22

**Authors:** Minggang Shao, Zhuhuang Zhou, Guangyu Bin, Yanping Bai, Shuicai Wu

**Affiliations:** 1 College of Life Science and Bioengineering, Beijing University of Technology, Beijing 100124, China; shaomg@emails.bjut.edu.cn (M.S.); zhouzh@bjut.edu.cn (Z.Z.); guangyubin@bjut.edu.cn (G.B.); baiyanping@bjut.edu.cn (Y.B.); 2 Smart City College, Beijing Union University, Beijing 100101, China

**Keywords:** electrocardiogram (ECG) monitoring, wearable ECG patch, atrial fibrillation detection, Android smartphone, cloud computing

## Abstract

In this paper we proposed a wearable electrocardiogram (ECG) telemonitoring system for atrial fibrillation (AF) detection based on a smartphone and cloud computing. A wearable ECG patch was designed to collect ECG signals and send the signals to an Android smartphone via Bluetooth. An Android APP was developed to display the ECG waveforms in real time and transmit every 30 s ECG data to a remote cloud server. A machine learning (CatBoost)-based ECG classification method was proposed to detect AF in the cloud server. In case of detected AF, the cloud server pushed the ECG data and classification results to the web browser of a doctor. Finally, the Android APP displayed the doctor’s diagnosis for the ECG signals. Experimental results showed the proposed CatBoost classifier trained with 17 selected features achieved an overall *F*_1_ score of 0.92 on the test set (*n* = 7270). The proposed wearable ECG monitoring system may potentially be useful for long-term ECG telemonitoring for AF detection.

## 1. Introduction

Atrial fibrillation (AF) is the most common sustained cardiac arrhythmia, affecting about 33.5 million people worldwide in 2010 [1]. There is growing evidence that AF is associated with sudden cardiac death, stroke, and congestive heart failure, etc. [2,3]. Early detection of AF is of critical importance.

AF detection is still challenging due to the fact that AF may be atypical or asymptomatic, especially in the elderly. As a result, guidelines from several professional associations recommend screening for AF by prolonged electrocardiogram (ECG) monitoring [4]. Traditional monitoring strategies with 12-lead ECG or Holter have a low detection rate due to the limited duration of the recording. Implantable cardiac monitor (ICM) supports long-term monitoring and is useful for detecting silent AF in patients. However, ICM is not suggested for screening AF because it requires invasive surgery. Recently, noninvasive long-term ECG monitoring has been the focus of research on screening methods for undiagnosed AF. Wearable long-term ECG monitoring systems using noninvasive sensors, including hand-held devices [5,6], skin patch recorders [7,8] and smart clothing [9], have been developed for the detection of undiagnosed AF. The wearable sensors usually have little impact on the user’s daily activities. With these systems, long-term ECG can be monitored for detecting undiagnosed AF. However, some systems using hand-held devices [5,6] restrict users’ mobility and the measuring time was usually limited to several minutes, which means the user’s ECG cannot be continuously monitored. Other systems [7,8] store the ECG data in the local storage, and therefore cannot support real-time monitoring. Furthermore, most existing systems [6,7,8,9] do not support online diagnosis or instant feedback.

Thanks to the popularity of smartphones and cloud computing, many researchers [10,11,12] have proposed a number of real-time telemonitoring systems based on cloud computing for healthcare or for detecting heart diseases. The cloud computing technology is a powerful tool to provide remote data transmission, complex computation, big data processing, and instant diagnosis. However, these cloud based telemonitoring systems still lack an algorithm for automatic AF detection.

An automatic AF detector can assist the doctors in identifying potential AF in a tremendous amount of ECG data. Therefore, an accurate AF detector is of clinical importance, especially in screening AF. AF detection methods using ECG signals are usually investigated from two aspects, the absence of P waves or the presence of f-waves [13,14], and irregularity of RR intervals [15,16,17,18,19]. In the 2017 PhysioNet/Computing in Cardiology (CinC) Challenge of AF classification (termed Challenge) [20], the official algorithms [21,22,23,24,25,26,27] based on machine learning, especially deep learning methods, have achieved excellent performance on AF detection.

The literature review reveals that the current ECG telemonitoring systems are not real-time or lack automatic AF detection. To this end, we proposed a near-real-time ECG telemonitoring system with automatic AF detection based on the smartphone and cloud computing in this work. The system could provide instant feedback from the doctor. We also improved the performance of our previous AF classification method [27] proposed in the Challenge. A wearable ECG patch device was designed to collect single-lead ECG signals and to continuously send the collected ECG data to an Android smartphone via Bluetooth. The Android APP displayed the ECG waveforms in real time and transmitted every 30 s ECG data to a cloud server. The cloud server used the improved ECG classification algorithm to detect AF and pushed the ECG data and classification results to the web browser of a doctor when AF was detected. Finally, the Android APP displayed the doctor’s diagnosis.

## 2. Materials and Methods

**Figure 1:**
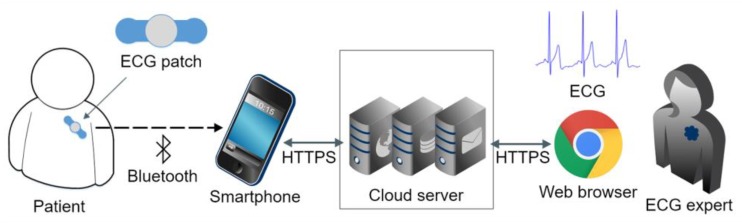
Illustration of the wearable ECG telemonitoring system.

The wearable ECG monitoring system comprises an ECG patch, an Android smartphone, and a cloud server (Figure 1).

**Figure 2:**
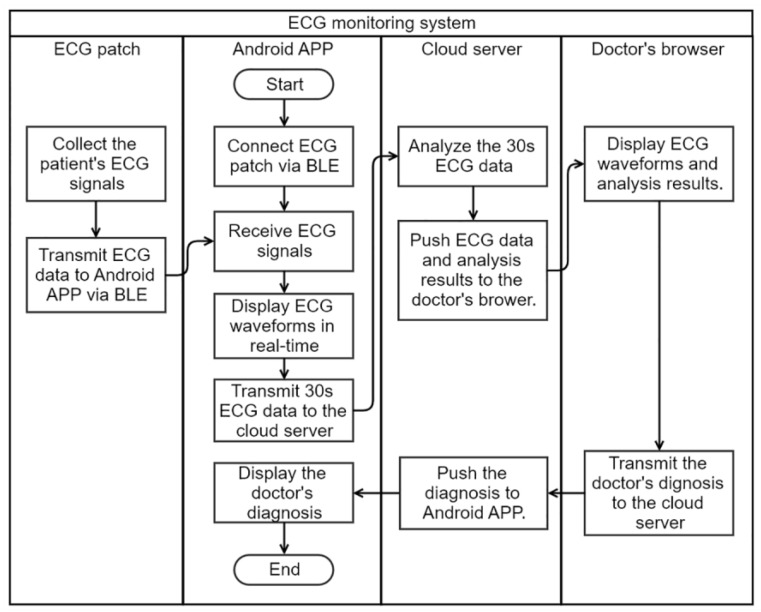
Flow chart of the proposed ECG monitoring System.

The flow chart of the system is shown in Figure 2. A wireless ECG patch device was designed to collect the user’s single-lead ECG signals, and transmitted the signals to the Android smartphone APP via Bluetooth Low Energy (BLE). The APP connected the ECG patch and started receiving the ECG data. The APP displayed ECG waveforms in real time, and transmitted every 30 s ECG data to the cloud server via HTTPS encrypted channel. In the cloud server, the 30 s ECG data were automatically analyzed by an AF detection algorithm; the ECG data and the analysis result were pushed to the web browser of the doctor when AF was detected by the algorithm. The doctor’s diagnosis was then pushed to the APP by the cloud server. Finally, the user could read the doctor’s diagnosis and would be advised to go to a hospital for review and management if necessary. 

### 2.1. Hardware Design of ECG Patches

**Figure 3:**
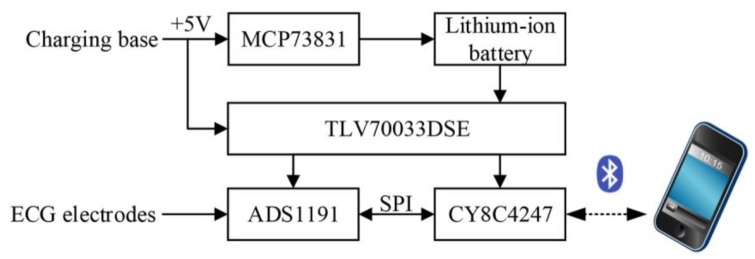
Block diagram of the hardware of the ECG patch.

**Figure 4:**
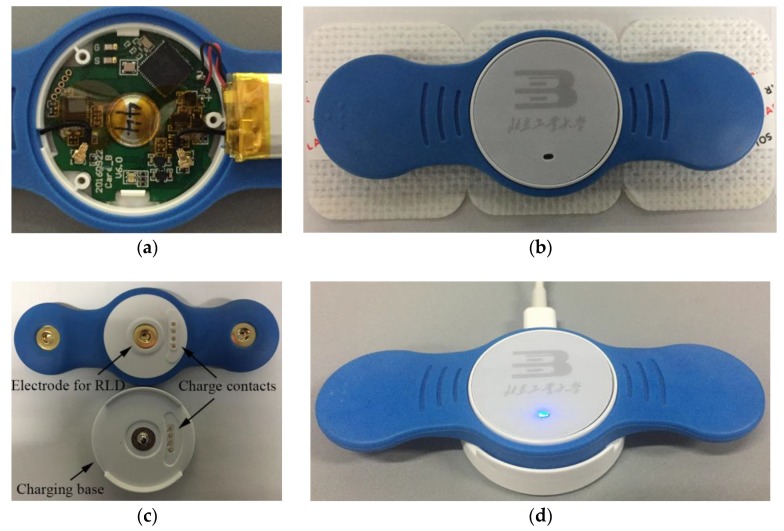
(**a**) The hardware circuit board with lithium-ion battery. (**b**) Front view of the ECG patch device with disposable ECG electrodes. (**c**) The three electrodes of the patch and four charge contacts of the patch and the charging base. (RLD = right leg drive). (**d**) The ECG patch was charging on the charging base.

The hardware of the patch was designed based on our previous work [28]. Figure 3 shows the block diagram of the hardware modules of the ECG patch, including a microcontroller supporting BLE 4.2, an analog front-end for signal acquisition, and a power-supply module with charge management. The microcontroller adopted CY8C4247 manufactured by Cypress Semiconductor Corporation (San Jose, CA, USA), which was a 48-MHz Arm Cortex-M0 CPU with a build-in BLE module. The analog front-end of ADS1191 from Texas Instruments Incorporated (Dallas, TX, USA) was selected for signal acquisition, which was a one-channel, 16-bit analog-to-digital converter for medical ECG measurement with built-in right leg drive (RLD) amplifier and lead-off detection. The power-supply module included a charge management controller (MCP73831 from Microchip Technology Incorporated, Chandler, AZ, USA), a low-dropout regulator of TLV70033DSE from Texas Instruments. An external charging base with micro USB port was used to provide +5 V power supply to the patch for charging the lithium-ion battery. The +5 V power supply was also used to supply power to the regulator of TLV70033DSE when charging. The output voltage of TLV70033DSE was fixed to 3.3 V for ADS1191 and CY8C4247. When the patch was docked on the charging base for charging, the patch stopped collecting signals and just blinked a LED to indicate the charging status. The built-in RLD amplifier of ADS1191 was used to design a RLD electric circuit for countering the common-mode interference [Figure 4c]. Lead-off detection was implemented in the patch to continuously monitor the electrode impedances to ensure reliable electrode connections. An LED of the patch was used to alert the user when a lead-off occurred. The sampling frequency was set to 250 Hz, and the patch continuously transmitted 200 ms ECG signals to the smartphone via BLE.

Figure 4a shows the hardware circuit of the ECG patch. The patch and its charging base are shown in Figure 4b and c. The charging base was used to charge the ECG patch [Figure 4d].

### 2.2. Android APP Software Design

The Android smartphone with the designed APP worked as a gateway between the ECG patch and the cloud server. The Android APP connected the ECG patch and continuously received ECG data. Then the APP displayed the ECG waveforms in real time, and transmitted every 30 s ECG data to the cloud server for near-real-time analysis. The APP also received and displayed the doctor’s diagnosis from the cloud. 

The Android Studio tool and the Java language were used to develop the APP software. The software modules of the APP were listed in Table 1, including a user module, a BLE module, a display and storage module, a historical data module, and a cloud module.

**Figure 5:**
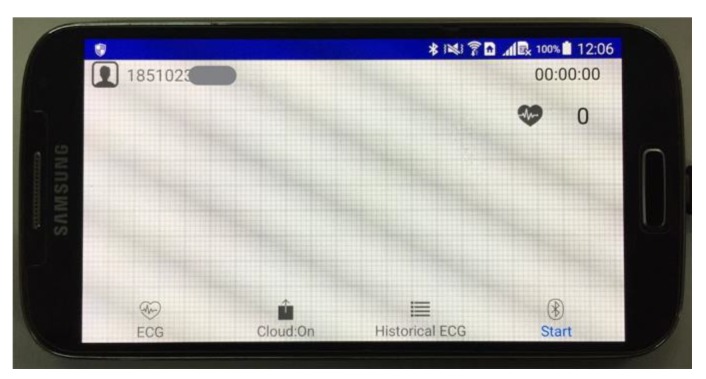
The main user interface of the Android APP.

The main user interface of the APP is shown in Figure 5. By clicking the “Start” button, the APP would try to connect ECG patch via BLE and receive ECG signals. ECG waveforms were displayed on the screen with heart rates shown. The “historical ECG” button was for reviewing the past ECG signals. The “Cloud: On/Off” button was used to enable or disable uploading ECG data to the cloud server.

### 2.3. ECG Classification Algorithm

During the Challenge, we proposed a novel ECG classification algorithm [27] using machine learning to classify a short single-lead ECG recording into one of four classes: Normal sinus rhythm, AF rhythm, Other arrhythmias, and Noisy recordings. In this work, we improved the performance of the previous algorithm from three aspects: (1) enhancing the dataset, (2) using CatBoost learning kit [29] to train a new classifier, and (3) selecting appropriate features to reduce the number of features without obviously decreasing the performance.

**Figure 6:**
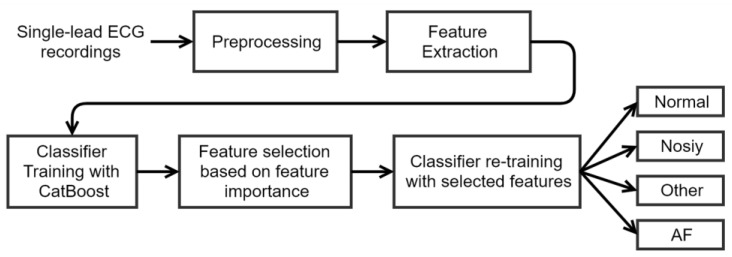
Flow chart of the ECG classification method proposed in this work.

Figure 6 shows the diagram of the method proposed in this work, including five steps: preprocessing, feature extraction, classifier training, feature selection, and classifier re-training. In preprocessing, the raw ECG signals were filtered with a bandpass Butterworth filter; R-peaks, RR and delta RR (dRR) intervals were calculated. In feature extraction, 31 multi-level features were extracted. In classifier training, a CatBoost model was trained with all the 31 features. In feature selection, features were selected according to the feature importance obtained in previous steps of classifier training. In classifier re-training, a CatBoost classifier was trained again with the selected features, and the obtained model was used to classify an ECG recordings into one of the four classes.

#### 2.3.1. Feature Extraction

A total of 31 features were extracted from each ECG recording, and the key features were depicted in Table 2. AF features were calculated based on RR intervals to measure the irregularity of RR intervals through the period of AF. Morphology features and RR interval features were related to many cardiac arrhythmias. Features for Noisy class were designed to recognize the Noisy class.

#### 2.3.2. ECG Classification

In this work, a decision tree ensemble classifier was trained using the CatBoost learning kit to classify an ECG recordings into one of the four classes. CatBoost is an open-source library for gradient boosting on decision trees and supports categorical features. 

The classifier training process included three steps. First, all the 31 features were used to train the CatBoost classifier. The training parameters are listed in Table 3. Secondly, feature importance was obtained from the trained CatBoost model. Features were ranked according to the feature importance. The top-importance features were used to evaluate the performance. At last, the selected features were used to re-train a final CatBoost classifier.

In this work, twenty-folds cross-validation was used during the training process. The training dataset was randomly split into twenty equal-size subsets. In a total of twenty training epochs, each subset was used as the validation set only once and the others as the training set.

#### 2.3.3. ECG Dataset

The Challenge provided an open ECG database (AFDB-2017) including 8528 single-lead ECG recordings lasting from 9 to 61 s [20]. Each recording was labeled to one of the four classes. Furthermore, the MIT-BIH Atrial Fibrillation Database (MITBIH-AFDB) [31] was utilized and the 23 recordings with raw ECG signals were used in this work. The 23 recordings contain two ECG signals, and only the first channel was used. The ECG data of each recording was split into 30 s segments. Each 30 s segment was labeled to one of the four classes according to the original rhythm annotation. The annotation of AFIB (i.e., atrial fibrillation) was labeled as AF class, AFL (atrial flutter) and J (AV junctional rhythm) as Other class, and N as Normal class. The annotations for the two databases used in this work were described in Table 4. 

The two databases were randomly divided into training and test sets. The major subsets of AFDB-2017 and MITBIH-AFDB were used as the training set (*n* = 29,074), and the minor subsets as the test set (*n* = 7270), as shown in Table 4.

### 2.4. Cloud Software Design

The cloud server was running as a gateway between the doctor and the Android APP, transmitting ECG data and doctor’s diagnosis between the Android APP and the doctor’s web browser. Besides, our AF classification method was deployed in the cloud server to analyze the ECG data.

The cloud server comprised three parts: web application programming interfaces (APIs), a web application of ECG diagnosis, and an ECG classification algorithm. Web APIs provided interfaces for the Android APP and the web application to access cloud data. The web application provided a browser-based user interface for doctors to diagnose the user’s ECG waveforms. The ECG classification algorithm was used to detect AF. 

The web APIs were designed using the Java language and hosted in a Tomcat container. HTTPS encrypted channel was enabled in Tomcat to ensure data security. The designed web APIs are shown in Table 5.

**Figure 7:**
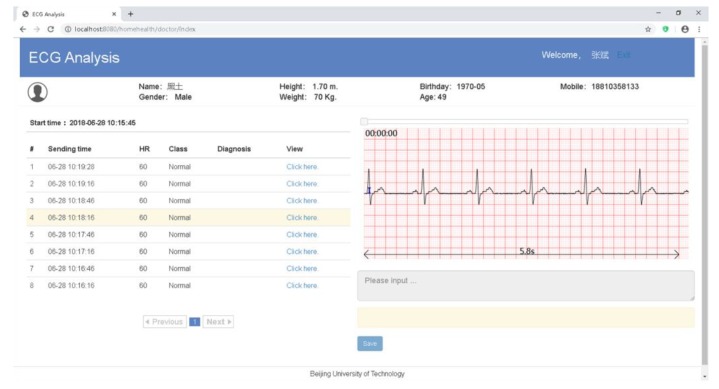
The main web page of the web application.

The main web page of the web application displayed the ECG waveforms and the analysis results on a web browser (Figure 7). When the “Save” button was clicked, the doctor’s diagnosis would be uploaded to the cloud. The web application was developed with HTML5 and the JavaScript language, and hosted in the same Tomcat container as the web API.

**Figure 8:**
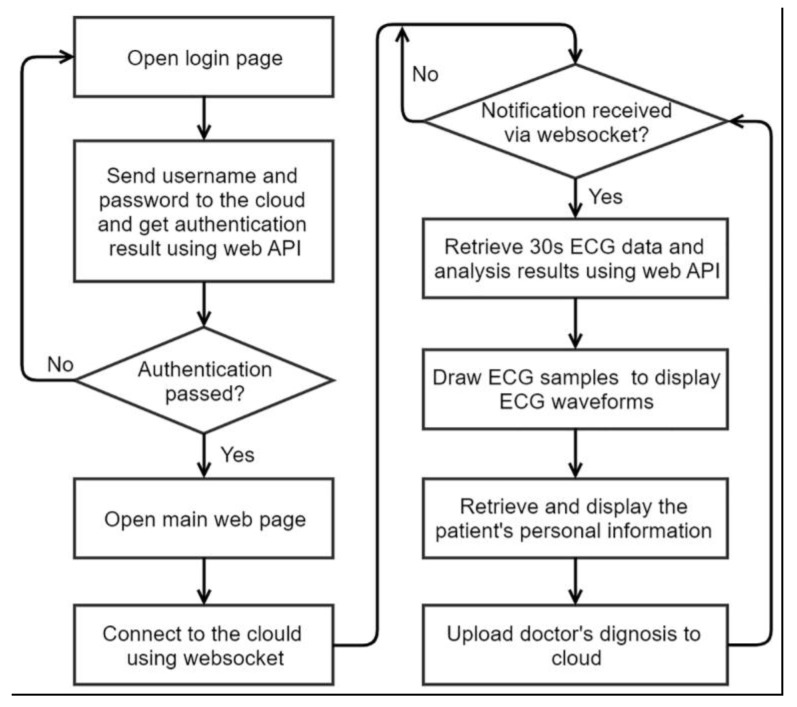
Flow chart of the web application of ECG diagnosis.

The flow chart of the web application is shown in Figure 8. The doctor opened the login web page using a web browser. The login page sent user name and password to the cloud and get the authentication result by accessing the web API. If authentication passed, the web browser would open the main web page. In the main web page, the websocket technology of HTML5 was used to establish a connection between the web browser and the cloud server to receive cloud notification in real time. When the cloud notification arrived, the browser would access the web API to retrieve the 30 s ECG data and the user’s personal information. Canvas of HTML5 was used to display ECG waveforms on the web browser. At last, the web browser uploaded the diagnosis to the cloud server. 

## 3. Results

### 3.1. Algorithm Perforcemence

**Figure 9:**
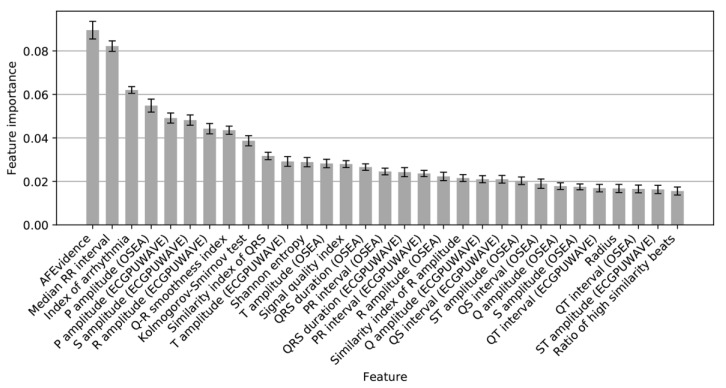
Illustration of the importance of the 31 features. (The top 17 features were selected to train the final classifier).

**Figure 10:**
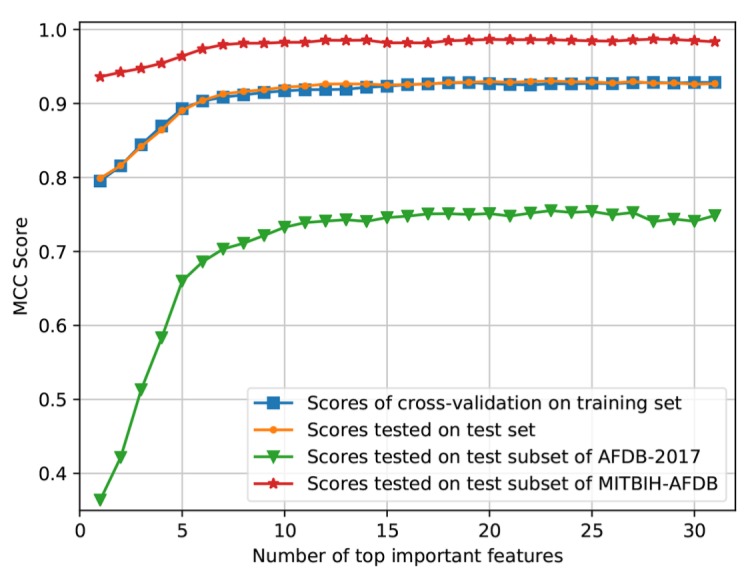
MCC scores according to different number of top-importance features. MCC = Matthews correlation coefficient.

As shown in Figure 9, 31 features were ranked by the feature importance, and the top-importance features were used to evaluate the performance of the CatBoost model. Figure 10 shows that the performance would not increase obviously from the number of 17. Accordingly, these top 17 features (Figure 9) were selected and used to re-train the final CatBoost model. The cross-validation scores of re-training and the test scores of the final model are shown in Table 6. The final CatBoost model achieved an overall *F*_1_ score of 0.92 on the test set.

The CatBoost model was also tested on the entire AFDB-2017 and MITBIH-AFDB datasets. For AFDB-2017, the *F*_1_ scores for Normal, AF, Other classes were 0.95, 0.90, 0.87, respectively, and the overall *F*_1_ was 0.91. For MITBIH-AFDB, when combining Normal, Other and Noisy classes as non-AF class, our AF detector achieved a sensitivity of 99.61%, a specificity of 99.64% and an accuracy of 99.62%. For the time efficiency of the proposed algorithm, the average processing time for a 30 s ECG segment was around 0.5 s.

### 3.2. System Verification

**Figure 11:**
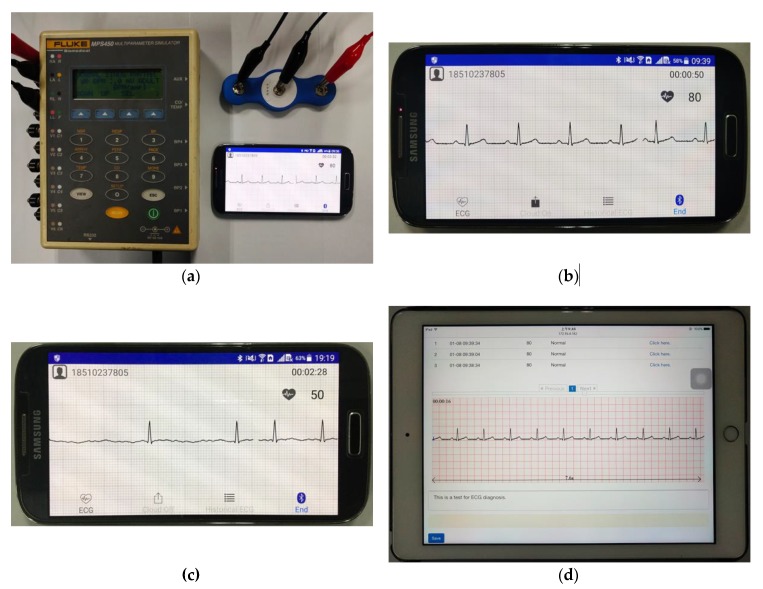

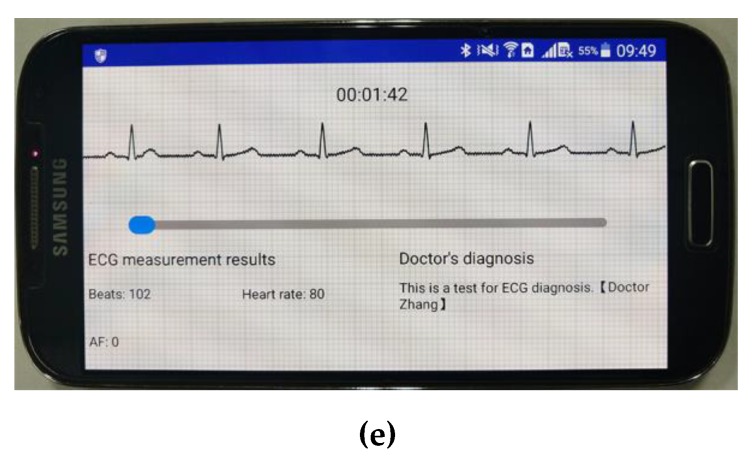
The ECG monitoring system in operation. (**a**) The ECG patch was connected to the FLUKE MPS450. (**b**,**c**) The Android APP displayed the normal (**b**) and abnormal (**c**) ECG waveforms in real time. (**d**) The doctor’s web browser displayed the 30 s ECG waveforms. (**e**) The historical data module of the APP showed the doctor’s diagnosis.

Simulated data were used to verify the proposed ECG monitoring system. As shown in Figure 11a, the ECG patch was connected to the ECG signal generator of FLUKE MPS450. The parameters to generate the ECG signals were 1.0 mV of amplitude, 80 BPM of heart rate and normal sinus rhythm [Figure 11b]. An abnormal ECG signal (AF) was also simulated by using MPS450 [Figure 11c]. The Android APP displayed the ECG signals in real time [Figure 11b,c]. The web browser of an iPad displayed the ECG signals pushed by the cloud server for testing, and the doctor’s diagnosis was saved to the cloud server [Figure 11d]. 

The ECG waveforms displayed on the Android APP were consistent with the parameters of FLUKE MPS450, and with the ECG waveforms displayed on the doctor’s web browser. These results demonstrated that ECG signal collection and transmission were reliable. The historical data module of the Android APP showed the ECG signals and the corresponding doctor’s diagnosis [Figure 11e]. These results showed that the proposed ECG monitoring system could work effectively.

## 4. Discussion

In this work, we proposed a wearable ECG telemonitoring system for AF detection based on smartphones and cloud computing. The AF classification algorithm based on machine learning achieved an overall *F*_1_ score of 0.92 on the test set. Experimental results showed that the system worked effectively, including ECG signals collection, real-time display of ECG waveforms, transmission of ECG data and ECG diagnosis. To the best of our knowledge, the system proposed in this study is the first near-real-time ECG telemonitoring system with automatic AF detection.

As shown in Figure 4, the ECG patch was designed to collect ECG signals. The patch was equipped with a 75 mAh battery, and would take about 60 min (40–80 min) to charge. The average working current was less than 3 mA. Hence, the patch can work continuously for 24 h, making it suitable for long-term and continuous ECG monitoring. With disposable ECG electrodes, the patch can be reused and is thus low-cost. The ECG patch device was compact and lightweight, and easy to wear on the chest. 

The ECG patch can continuously transmit the ECG signals to the smartphone via Bluetooth. The smartphone APP software can display the ECG waveform in real time and upload every 30 s ECG data to the remote cloud server. The cloud server uses the AF classification algorithm to classify the 30 s ECG data into one of the four classes, and push the ECG data and classification results to the web browser of the doctor when AF is detected. Finally, the user can read the doctor’s diagnosis and will be advised to go to a hospital for review and management if necessary, with AF reports provided to the patient’s physician in the hospital when requested. Furthermore, a telephone follow-up for the patient will be performed to track the diagnosis and treatment of AF. The proposed monitoring system has the advantage of mobility, which means that the users can be monitored anytime and anywhere by using a smartphone, and the doctors can make diagnosis of the user’s ECG anytime and anywhere by using cloud computing.

ECG signals can be distorted by artifacts caused by physiological and non-physiological sources, such as muscular activity, patient motion, electromagnetic interference, and cable and electrode malfunction [35]. At present, the signal quality was ensured by two ways. First, lead-off detection implemented in the patch was used to ensure suitable electrode connections. Second, if the 30 s ECG signals transmitted to the cloud by the user’s smartphone was too noisy to analyze, the support staff would call the user and guide the user to wear the patch correctly. Usually, careful guidance may address most of issues related to unsuitable signal quality, making adequate ECG analysis possible.

This work also presented a multivariate method based on machine learning for AF detection. A comparison with the top algorithms from the Challenge is listed in Table 7. The algorithms were all based on machine learning methods including decision tree ensemble (DTE) and deep neural network (DNN). Teijeiro et al. [23] and Hong et al. [22] combined conventional machine learning and deep learning methods to achieve better performance. The algorithms of Zihlmann et al. [26] and Xiong et al. [24] were based on DNN, which is an end-to-end process without extracting hand-crafted features. Using only 30 features, our previous method [27] proposed in the Challenge achieved an overall *F*_1_ score of 0.87 on AFDB-2017. In comparison with our previous method, the CatBoost model proposed in this work achieved an overall *F*_1_ score of 0.91 on AFDB-2017, which increased by 0.04, while using less features. It is also worth noting that the algorithms of Datta et al. [21], Zabihi et al. [25] and Hong et al. [22] achieved high performance on the training set but much lower performance on the test set. The reason may be the overfitting problem was not prevented when training the models. In this work, as shown in Figure 10, the scores on the test set were close to those on the training set and the overfitting problem was suppressed by selecting appropriate training parameters.

Table 8 compares our method with some of the most-cited and recent AF detectors on MITBIH-AFDB. These algorithms were based on P wave analysis [13], RR interval irregularity [15,16,17,18,19], the combination of RR interval and P wave analysis [36], or wavelet [37]. The approach of Jiang et al. [36] was based on the combination of RR interval and P wave analysis, and achieved a sensitivity of 98.2% and a specificity of 97.50%. The algorithms of Asgari et al. [37], which were based on stationary wavelet transform and support vector machine, have the advantage of no need to detect beats. Our method achieved the highest performance of sensitivity = 99.61%, specificity = 99.64% and accuracy = 99.62%, mainly due to the fact that the 17 selected features (Figure 9) were important and efficient to detect AF, including AFEvidence, P amplitude (OSEA), P amplitude (ECGPUWAVE), Kolmogorov–Smirnov test, and Shannon entropy features.

Comparing Table 7 and Table 8, we may find that the performance of most algorithms on AFDB-2017 was much lower than that on MITBIH-AFDB. Figure 10 also shows that the MCC scores on the test subset of AFDB-2017 are much lower than those on the test subset of MITBIH-AFDB. One plausible reason is the ECG signals of AFDB-2017, which were collected using wearable ECG devices, have much noise contamination and low resolution compared with the signals of MITBIH-AFDB. Another reason may be that the annotations of MITBIH-AFDB are mainly Normal class and AF class (Table 4), while AFDB-2017 includes many ECG recordings of Other class (Table 4). As demonstrated in the Challenge, many non-AF rhythms present irregular RR intervals similar to AF [20], making it difficult to distinguish between Other class and AF class. Our CatBoost model misclassified a certain number of ECG recordings of Other class to AF class, resulting in that the *F*_1_ score of Other class was much lower than those of the Normal and AF classes (Table 6).

The CatBoost classifier used in this work improved the previous method from three aspects. (1) The datasets were enlarged using MITBIH-AFDB and AFDB-2017. (2) A new classifier was trained by using the CatBoost learning library and achieved an overall *F*_1_ metric of 0.92 on the test set. (3) 17 features were selected by using feature importance, and the classifier trained with these 17 features achieved superior performance.

The AliveCor heart monitor (AliveCor, Inc., Mountain View, CA, USA) has been used to collect ECG signals for AF detection [5,6]. The ECG data can be transmitted to a smartphone in real time and the users may receive the diagnosis in 24 h. The disadvantage of the AliveCor heart monitor was the short measurement time which was limited to a few minutes for each measurement. AliveCor cannot be used to monitor the user’s ECG continuously. Thus, the users with atypical AF may be undetected. Turakhia et al. [7] and Steinhubl et al. [8] used a wearable continuous ECG monitoring patch to identify AF. This patch was small-sized and water-proof. It stored the collected ECG data in the local storage, and can be used only once. After a measurement period of two weeks, the users delivered their patches to the doctors and get diagnosis results later. The users cannot be monitored in real time. Fukuma et al. [9] evaluated the feasibility of a wearable ECG monitoring with T-shirt embedded in fabric electrodes for AF detection in young people. The T-shirt with fabric electrodes is comfortable to wear and can prevent skin erosion. The collected ECG signals were transferred to a remote data server and manually analyzed by cardiologists. However, the tight-fitting T-shirt was not suitable for the elderly or for continuous monitoring. With the advantage of cloud computing, the cloud based telemonitoring systems [10,11,12] supports real-time monitoring, automatic heart disease detection, and instant feedback, but there is no algorithm implemented for detecting AF. Compared to these monitoring systems, the wearable ECG monitoring system developed in this work has the following advantages. (1) The patch designed in this work supported continuous monitoring and can be reused at low cost. (2) Real-time ECG monitoring was implemented by using the smartphone and cloud computing, with near-real-time analysis and instant feedback. (3) An automatic AF detector was implemented to assist doctors in identifying potential AF in patients.

This work has several limitations and improvements may be considered in future work:

(1) At present, the system was only tested with open-access ECG databases. Clinical studies may be conducted in future work.
(2) An ECG quality assessment method may be developed and implemented in the smartphone. The quality assessment method could automatically estimate the signal quality in real time, and the user would be alerted when the signal quality is too low.
(3) Currently, when a 30 s ECG segment is detected as AF by the proposed algorithm, all the ECG signals will be transferred to the doctor for diagnosis. However, the algorithm may have false-negative detections of AF. Some improvements may be considered in future work, including (a) improving the performance of the AF detector, especially reducing false-negative detections; (b) an index for AF detection can be provided for doctors so that the doctors can adjust the false negative and false positive rates by selecting different values of the index; (c) statistical analysis can be conducted on the clinical data collected by the proposed system for estimations of false positive and false negative rates, with causes for misdetection analyzed.

Deep learning, like recurrent neural network (RNN) and convolutional neural network (CNN), was promising methods for ECG classification [24,25,26]. The traditional machine learning methods, such as XGBoost, CatBoost, bagged or AdaBoosted decision tree ensemble, and support vector machine, combined with deep learning methods may be useful to improve the performance of AF detection [23]. The hybrid method may be investigated in future work.

## 5. Conclusions

A wearable ECG monitoring system based on the smartphone and cloud computing was proposed in this work. An ECG classification algorithm for AF detection based on machine learning was used to classify the ECG signals into one of the four classes. An ECG patch device was designed to collect the user’s ECG signals. An Android APP was developed to display ECG waveforms in real time. Every 30 s ECG data were transmitted to a remote cloud server, and classified to one of the four classes by the proposed algorithm. A browser-based ECG diagnosis tool was developed for doctors. Experimental results showed that the system worked effectively and the ECG classification algorithm achieved an overall *F*_1_ score of 0.92. The proposed wearable ECG monitoring system may potentially be useful for long-term ECG telemonitoring for AF detection.

## Figures and Tables

**Table 1:** Descriptions of the software modules of the APP.

Module	Description
User module	Register user information to the cloud and log in the APP with registered mobile number and password
BLE module	Connect the ECG patch via BLE and receive ECG data continuously
Display and storage module	Display ECG waveforms and store the raw ECG data to the internal storage in real time
Historical data module	Display historical ECG waveforms and the related doctor’s diagnosis
Cloud module	Transmit 30 s ECG data to the cloud and receive the doctor’s diagnosis

**Table 2:** Description of the key features.

Group/Feature		Description
AF features		
	AFEvidence [17]	AFEvidence feature was calculated based on the two-dimensional histogram built on the Lorenz distribution of dRR intervals from the whole ECG recording.
	Shannon entropy [30]	Shannon entropy feature was computed on the histogram of the dRR intervals.
	Kolmogorov–Smirnov test [15]	Kolmogorov–Smirnov test feature was obtained by evaluating the difference between the distribution of the ECG recording and the reference distribution for AF.
Morphology features		Ten kinds of Morphology features were extracted by using two open source libraries, the ECGPUWAVE [31] and the OSEA [32], respectively, including (1) QRS duration, (2) PR interval, (3) QT interval, (4) QS interval, (5) ST amplitude, (6) P amplitude, (7) Q amplitude, (8) R amplitude, (9) S amplitude, and (10) T amplitude.

RR interval features		
	Median RR interval	Median RR interval feature was the median value of all RR intervals extracted from the entire ECG recording.
	Index of arrhythmia [33]	Index of arrhythmia feature was the number of abnormal beats in an ECG recording. The abnormal beats were determined by four knowledge-based conditions. These conditions were judged according to three continuous RR intervals and their mean value.
Features for Noisy class		
	Similarity index of QRS	Similarity index of QRS was the mean value of correlation coefficients calculated between every two QRS waveforms from the whole ECG recording.
	Signal quality index	Signal quality index represented the ratio of high signal quality beats in an ECG record. A high signal quality beat was decided by evaluating the amplitudes of the isoelectric level.
	Q-R smoothness index(QRsi)	QRsi feature represented the smoothness of the segment from the average beat. The segment was the increased QRS amplitude lasting from QRS onset to R-peak. QRsi value was defined as the peak numbers computed on the difference values of samples.

**Table 3:** Parameters for training the CatBoost model.

Parameter	Value
learning_rate	0.1
Iterations	276
early_stopping_rounds	20
depth	8
l2_leaf_reg	3
bagging_temperature	0.7
random_strength	0.2
leaf_estimation_method	“Newton”
random_seed	(Random integer)
loss_function	MultiClass
eval_metric	Matthews correlation coefficient (MCC) [34]

**Table 4:** Annotations for the two ECG databases.

Database	Training Set	Test Set	Annotation				Total
			Normal	AF	Other	Noisy	
AFDB-2017	6822	1706	5076	758	2415	279	8528
MITBIH-AFDB	22,252	5564	16,554	11,066	196	0	27,816
Total	**29,074**	**7270**	21,630	11,824	2611	279	**36,344**

**Table 5:** Descriptions of the web APIs.

Used by	URL of Web API ^1^	Description
Android APP	/user/register	Receive user information including mobile number and password, and store them in a database.
	/user/login	Receive mobile number and password from Android APP, determine the correctness and return the authentication result.
	/user/ecgsegment	Receive 30 s ECG data, analyze it using the ECG classification algorithm, save the data and the analysis results in a database, and send a notification to the web application.
	/user/ecgdiagnosis	Provide the ECG diagnosis requested by the Android APP.
Web application of ECG diagnosis	/doc/login	Receive the doctor’s username and password from the web browser, and determine the validity.
	/doc/userinfo	Transmit the requested user’s personal information to the web browser.
	/doc/ecgsegment	Transmit the requested 30 s ECG data to the web browser.
	/doc/ecganalysis	Receive the doctor’s diagnosis, save it in a database and send a notification to the Android APP.

^1^ The URL started with https://<domain name or IP address>, and the domain name or the IP address of the URL were omitted which were decided by the cloud server.

**Table 6:** Description of cross-validation scores and test scores.

Classifier	Testing Set	No. of Cases	Acc	*F*_1n_	*F*_1a_	*F*_1o_	*F*_1_
Cross-validation	Training set	29,074	0.96	0.98	0.98	0.79	0.92
CatBoost model	Test set	7270	0.96	0.98	0.98	0.80	**0.92**

Acc = accuracy score; *F*_1n_ = *F*_1_ score of Normal class; *F*_1a_ = *F*_1_ score of AF class; *F*_1o_ = *F*_1_ score of Other class; *F*_1_ = scoring metric of the Challenge, which is an average of *F*_1n_, *F*_1a_ and *F*_1o._

**Table 7:** Performance comparison of AF classifiers on the Challenge dataset.

Authors	Algorithm	No. of Features	Training Set (AFDB-2017) ^1^				Test Set ^1^
			*F*_1n_	*F*_1a_	*F*_1o_	*F*_1_	*F*_1_
Teijeiro et al. [23]	XGBoost and DNN	79	0.94 ^2^	0.90 ^2^	0.84 ^2^	0.89 ^2^	0.83 ^2^
Datta et al. [21]	DTE	150	0.99 ^2^	0.94 ^2^	0.98 ^2^	0.97 ^2^	0.83 ^2^
Zabihi et al. [25]	DTE	491	0.98 ^2^	0.93 ^2^	0.95 ^2^	0.95 ^2^	0.83 ^2^
Hong et al. [22]	DNN and XGBoost	300	0.99 ^2^	0.94 ^2^	0.98 ^2^	0.97 ^2^	0.83 ^2^
Zihlmann et al. [26]	DNN	-	0.93 ^2^	0.91 ^2^	0.83 ^2^	0.89 ^2^	0.82 ^2^
Xiong et al. [24]	DNN	-	0.93 ^2^	0.88 ^2^	0.83 ^2^	0.88 ^2^	0.82 ^2^
Our previous work [27]	DTE	30	0.93 ^2^	0.88 ^2^	0.82 ^2^	0.87 ^2^	0.82 ^2^
This work	CatBoost	17	0.95	0.90	0.87	**0.91**	-

^1^ The datasets of the Challenge; test set is unavailable to the public. ^2^ Official scores of the Challenge (https://physionet.org/content/challenge-2017/1.0.0/results_all_F1_scores_for_each_classification_type.csv). XGBoost = eXtreme gradient boosting; DTE = decision tree ensemble; DNN = deep neural network.

**Table 8:** Performance comparison of AF detectors on MITBIH-AFDB.

Algorithm	Sensitivity (%)	Specificity (%)	Accuracy (%)
Slocum et al. [13]	62.80	77.46	-
Tateno and Glass [18]	94.40	97.20	-
Sarkar et al. [17]	97.50	99.00	-
Huang et al. [15]	96.10	98.10	-
Lee et al. [16]	98.20	97.70	-
Jiang et al. [36]	98.20	97.50	
Zhou et al. [19]	96.89	98.25	97.67
Asgari et al. [37]	97.00	97.10	97.10
This work	99.61	99.64	99.62
